# Research on the flow experience and social influences of users of short online videos. A case study of DouYin

**DOI:** 10.1038/s41598-023-30525-y

**Published:** 2023-02-27

**Authors:** Cheng Zheng

**Affiliations:** Art Design College, Yangzhou Polytechnic Institute, Yangzhou, China

**Keywords:** Physiology, Psychology, Environmental social sciences

## Abstract

Recently, short online videos have been highly recognized by video market users and have developed rapidly. This study aims to explore why users enjoy watching and sharing short online videos by applying the theory of flow experience. Previous research has extensively examined traditional video arts such as TV and movies and text or image based, while research on short online videos has increased only in recent years. To improve the precision and comprehensiveness of the research, social influence is also used as a variable. This study takes the short video representative platform DouYin as a case study and the Chinese user market as the background. Through questionnaires, 406 users' data about short online video experiences were collected. After statistical analysis, the study finds that flow experience has a significant impact on participative behaviour and sharing behaviour for short online videos. According to further analyses, the flow experience, social norms, perceived critical mass and participative and sharing behaviour constitute three groups of mediating relationships. Finally, the discussion of the research results provides help to broaden the academic scope of the flow experience and video art, improve the short online video platform environment, and upgrade short online video services.

## Introduction

Currently, people's demand for image art does not stop at traditional film and television. Thanks to the development of network communication and large screen smartphone technology, the network short video market with mobile phones as terminals has been expanding rapidly. According to the 49th Statistical Report on the Development of China's internet released by the China Internet Network Information Centre (CNNIC), as of December 2021, the number of short video users had reached 934 million, with a utilization rate of 90.5%. In contrast to traditional movies and televisions, short video platforms are dedicated to creating a flow experience for users; this allows them to compete for the market, innovatively collect and analyse users’ behaviour data in the process of watching short videos, and constantly push their favourite videos to users. DouYin is a representative short video platform worldwide that has a strong user base due to its core technology of data push. DouYin released the DouYin and User Research Report in April 2018, which showed that approximately 22% of users used the platform more than one hour a day, and the proportion of daily active users and monthly active users reached 0.45. In contrast, the proportion of activity produced by the flow experience of the game was usually only between 0.3 and 0.6. Each person used the short video app for more than 22 h per month, with an average of 45 min per day. According to iMedia Research, 37.2% of DouYin users spent more time on the platform in 2021. The 2021 DouYin Data Report states that in the second half of 2021, DouYin successively launched a variety of functions to remind users to use mobile phones appropriately.

The previous literature has rarely involved theoretical research on the flow of short online videos, including the flow experience in the field of social networking. Therefore, this research takes the Chinese short online video user market as the background, adopts quantitative research method and collect data in the form of user questionnaires to explore the following questions:

- Is the flow experience related to the prevalence and dissemination of short online videos? What role does the flow experience play?
- Which variables are related to users' continuous viewing and dissemination of short online videos? What kind of relationship do they have?
- What is the relationship between the social influence involved in this study and other variables?

## Literature review

### Short online video

Short online video in this study refers to video content that is suitable for viewing on mobile phones and in shortterm leisure states and that is played, shared by various network new media platforms, and pushed with high frequency, ranging from a few seconds to a few minutes^1,2^. It covers sports, cooking, film, education, health, science and technology. Although the length is generally no more than 15 s, the content of short videos is very diverse. Users follow and interact with each other by watching, liking, commenting on and sharing videos^3^. Taking the Chinese market as an example, People's Daily Online (2020) defines short online videos as a form of internet content dissemination that is generally a video that is spread on new internet media within 5 min^4^.

In 2011, the mobile short video social app Viddy was publicly released, which was the first mobile application in the world that could publish and share short videos within 15 s^5^. In 2013, Twitter officially launched the Vine iOS version of its video sharing application, which allowed users to shoot 6-s video clips and seamlessly embed them into Twitter messages. In the same year, Instagram, an image social application with more than 100 million users, also launched a video sharing function with a video capture length of 15 s^6^.

The Musical.ly, short video music community application went online in April 2014. Users are able to quickly create a 15 s music video by matching their own videos with music in the music library or selecting their favourite popular songs, and they can produce music videos through mouth and body movements.

DouYin short online video is a representative platform in the industry of China. DouYin, which was originally music based creative short video social software, was launched on September 20, 2016. It was positioned as a short music video community platform for all ages.

Because the early functions of Musical.ly and DouYin were highly consistent, in December 2017, Musical.ly was acquired by Beijing ByteDance Company, and in 2018, users were transferred to TikTok, the overseas version of DouYin^7^.

Currently, DouYin is only for Chinese users, while TikTok is for international short video social apps in more than 150 countries and 75 languages. TikTok has many young users, and the content is mainly shared by these young people on a daily basis, with native content as the mainstream. However, DouYin covers all ages in China.

After development, DouYin has become a short video platform with multiple contents and rich user levels. It mainly adopts picture sequence playing, video instant shooting, and video uploading with match text and music for video presentation to users. The platform supports MP4 and WEBM video format upload. The viewing process is very simple and fast, users use finger to double click means Like, single click means continue or pause, pull screen down for switching to the next video, slide left to view the user's home page. On the top right corner of the interface, there is a search function. On the right side of the interface, there are functions for collection, comment and sharing. At the same time, DouYin has been committed to upgrading and simplifying its software operation functions and to, reducing the professional level and technical requirements of ordinary users. Its social attributes are very obvious. Users can easily share and spread media on social platforms after shooting and editing them on mobile phones or professional devices.

### Flow experience

In the 1960s, the concept of flow was first proposed by Mihaly Csikszentmihalyi, a Hungarian psychologist. His view on the theory of flow was based on the observation that painters can ignore hunger, fatigue and various external disturbances when they are engrossed in their creations. To study the intrinsic motivation of this kind of driving behaviour, he proposed flow theory and performed a systematic study. The early research of Csikszentmihalyi mainly focused on the leisure field, such as game research (1971)^8^, rock climbers, chess players, athletes and artists (1975)^9^. At the end of the twentieth century and the beginning of the twenty-first century, flow theory was developed in the context of the rapid progress of information technology. Scholars represented by Hoffman^10^ and Novak^11^ transformed the flow behaviour of focusing on browsing the web into a cognitive state during the process of online information acquisition. At the same time, they applied the theory of flow to online learning for the first time. Through the constructed network flow model, they proposed that the flow experience makes learning more exploratory and has a positively subjective experience. Therefore, research on internet behaviour based on flow theory has been subdivided into e-mail^12^, online consumer activity^13^, and mobile instant messaging^14^. Huang^15^ thought that the basic factors of network flow experience could be summarized as curiosity, control, internal interest and attention, which have been used to measure the flow experience behaviour of network users^15^. The popularity of online games also makes some scholars in the field of flow theory focus on online game addicts.

## Conceptual framework and hypothesis development

### Participative behaviour and sharing behaviour of short online videos

For this study, in the process of using DouYin to watch short videos, users' information behaviour was divided into participative behaviour and sharing behaviour.

In this study, participative behaviour of short online videos has the following definitions:

- For short videos that users like to watch, users will collect them;
- Some short videos that are approved by users or cause emotional resonance will be praised;
- Some short videos will arouse users' interest and be commented on by users.

In contrast, the sharing behaviour of short online videos involved in this study has the following definitions:

- Users take their own short online videos and upload them to the short video platform online;
- Short online videos taken by users themselves are shared through the network social circle;
- When exposed to some short videos that are highly recognized, users will share these videos in their own network social circles and express their views or opinions, spreading their ideas.

Regarding the information recommended by the platform, users examine information and follow the information that arouses their interest. For DouYin, users just watch part of the video content and only make comments as well as praise and collection decisions for interesting video content or authors. Any action of the actors is not isolated but interrelated. The relationship between them is the channel of information and resource transmission, and the network relationship structure determines their action opportunities and results^16^. From the perspective of communication, when highly accepted video content is followed, this leads to affirmation of the quality of information content and emotional identification and, finally, sharing with people and society. Xue and Xu noted that information sharing promotes more information sharing to achieve widespread information^17^. Accordingly, the following hypotheses are proposed:

#### H1

Participative behaviour in short online videos has a significant positively effect on sharing behaviour.

### Social influence

Users' decisions about online media are often influenced by social circles, not just individual choices. From the perspective of social psychology and economics, social influence is divided into social norms and perceived critical mass. The former means that the members of a social circle will abide by the group norms, and the group norms will act on the members themselves. The latter means that when the internal and external influence of the network platform reaches a critical point, the number of network platforms will increase significantly, which will affect people's acceptance of the network platform. Cheung et al.^18^, Straub^19^, Liao et al.^20^, Liker and Sindi^21^, Hsu and Lu^22^ all made hypothetical relations between social influence and human behaviour. For short videos, social groups pay the same attention to the same kind of short videos, exchanging and sharing with each other. This kind of behaviour comes from social identity, which refers to the behaviour by which people receive incentives to maintain the self image of group members^23,24^. Moreover, people with a strong sense of mutual identity are more likely to recognize their identity within their social circle and to distinguish characteristics outside their social circle, actively increasing their image in the social circle^24^. In other words, DouYin is not only a short video viewing platform; users also use the social functions of DouYin to comment on, collect and share short videos. Yunwen Wang^3^ noted that for influencers of social media and their followers, short online videos form a quasis ocial bond and promote creativity, services and the sales of products^3^. Accordingly, the following hypotheses are proposed:

#### H2

Social norms have a significant positive effect on the participative behaviour of short online videos.

#### H3

Social norms have a significant positive effect on the sharing behaviour of short online videos.

#### H4

Perceived critical mass has a significant positive effect on the participative behaviour of short online videos.

#### H5

Perceived critical mass has a significant positive effect on the sharing behaviour of short online videos.

#### H6

Participative behaviour in short online videos plays a mediating role between social norms and sharing behaviour.

#### H7

Participative behaviour in short online videos plays a mediating role between perceived critical mass and sharing behaviour.

### Flow experience and short online videos

Chou and Ting^25^ proposed that people who have experienced flow are more likely to indulge in online games^25^. Chang and Zhu demonstrated that flow on social networking platforms significantly affects user satisfaction and continuous use intention^26^. Jung, Perez Mira and Wiley Patton proposed that for mobile TV, flow theory has a significant impact on consumers' acceptance of modern technology products^27^. The above research shows that flow theory is a kind of psychological process that can affect users' perception, attitude and communication of online social media from multiple dimensions and can ultimately affect the results. Therefore, this study further speculates that for the short online video platform of DouYin, the psychological effect generated by the flow experience affects users' preference for the short videos they watch, and they share videos with emotional resonance.

Accordingly, the following hypotheses are proposed:

#### H8

The flow experience has a significant positive effect on the participative behaviour of short online videos.

#### H9

The flow experience has a significant positive effect on the sharing behaviour of short online videos.

#### H10

Participative behaviour in short online videos plays a mediating role between flow experience and sharing behaviour.

### Platform interactivity

Alan Cooper suggested that the design of interactivity originated from the study of artificial interfaces, which provide a simple and easy interface to enable products to be recognized^28^. Therefore, in this study, the platform interactivity of short online videos should have the following definitions:

- Perceived interactive ability between the platform and users so that users can always find what they want to see;
- Short online video platforms can provide a good application interface to achieve information exchange between the platform and users or among users. The former refers to the platform providing users with an active environment for processing information and providing timely feedback on the instructions issued by users, while the latter refers to users having a good information exchange interface;
- Short online video platforms can provide users with a better viewing system so that they can make informational instructions for visual adjustment and other information exchange activities while viewing.

Blattberg and Deighton^29^ believed that interactivity should be understood as an information exchange between individuals who not constrained by distance or time^29^. For short online video platforms with social attributes, for example, information exchange is a series of interactions on DouYin among short video authors and appreciators and between users and platforms through search, comment, praise, collection, and subscription. Su and Hsaio^30^ took interactivity as a variable in their research on game learning systems and flow experience^30^. Previous studies by scholars including Hoffman and Novak^31^, Novak et al.^11^, Skadberg and Kimmel^32^, and Choi et al.^33^ also noted that there is a relationship between interactivity and flow experience. For DouYin, the relationship between the effect of flow experience and participative behaviour is affected by platform interactivity.

Accordingly, the following hypotheses are proposed:

#### H11

Platform interactivity moderates the link between the flow experience and participative behaviour of short online videos.

## Methods

### Data collection

The questionnaire design of this study aimed to explore the role of flow experience in the process of users' continuous viewing of short online videos and to more accurately identify the relationship between flow experience, user behaviour and social influence. The questionnaire was designed by Questionnaire Star software for the Chinese user market and randomly distributed among the author's personal contacts. The data were collected by the social networking software WeChat to users who had experience with DouYin short video In January 2022. SPSS version 24 and PROCESS were applied to analyse the study.

In this study, 450 questionnaires were conducted for two weeks. A total of 406 valid questionnaires were collected with an effective rate of 90.2%, of which 45.1% were male (n = 183) and 54.9% were female (n = 223). The proportion of respondents aged 18–30 and over 45 was high, at 43.1% (n = 175) and 34.2% (n = 139), respectively. A total of 70.9% (n = 288) of the respondents were from second and third tier cities the lowest proportion of first-tier cities was 11.1% (n = 45). Furthermore, 89.4% of the respondents had a college education or above, and 8.9% (n = 36) had a highest degree of Ph.D. The profile of the respondents is summarized in Table 1.

**Table 1:** Profile of respondents.

Measure	Items	Frequency	Percentage
Gender	Male	183	45.1
	Female	223	54.9
Age (years)	< 18	32	7.9
	18–30	175	43.1
	30–45	60	14.8
	> 45	139	34.2
City	Tier-1	45	11.1
	Tier-2	130	32
	Tier-3	158	38.9
	Tier-4 and below	73	18
Education	High school or less	43	10.6
	Some college or bachelor’s degree	261	64.3
	Master's degree	66	16.3
	Doctoral degree	36	8.9

### Measurement

All the questions in the Online Appendix S1 were developed from previous studies. The items on platform interactivity referred to Chang^34^ and Lee^35^. The items on flow experience were assessed based on Chang^34^. The relevant research of Zhou and Lu^14^; and Chen and Wang^36^ provided references for the items on participative behaviour and sharing behaviour. The items on social norms and perceived critical mass were assessed based on Hsu and Lu^22^. Ten constructs of 18 items were measured by a 7-level Likert scale from 1 (strongly disagree) to 7 (strongly agree).

### Ethics statement

All experimental protocols in this study were approved by the ACADEMIC COMMITTEE of YANGZHOU POLYTECHNIC INSTITUTE, which has the functions of academic ethics evaluation and supervision, including any relevant details. All methods were carried out in accordance with relevant guidelines and regulations.

The data involved in the study were collected in the form of an online questionnaire. All participation was voluntary, and written informed consent was obtained from all subjects or their legal guardians. The procedure was approved by the ACADEMIC COMMITTEE of YANGZHOU POLYTECHNIC INSTITUTE.

## Results

### Measurement model

First, confirmatory factor analysis (CFA) was conducted on 6 dimensions of the model to test the validity of the research model, including composite reliability (CR), average variance extracted (AVE), convergent validity and discriminatory validity. Confirmatory factor analysis (CFA) is a statistical technique used to verify the factor structure of a set of observed variables. CFA allows researchers to test the hypothesis that there is a relationship between observed variables and their potential structure. Researchers use theoretical knowledge, empirical research or both to presuppose the relationship model and then test the hypothesis statistically^37^.

Based on the factor loading results, credibility (Cronbach's alpha), composite reliability (CR), average variance extracted (AVE), convergent validity and discriminant validity were tested.

The results in Table 2 illustrate that the factor loadings of all indicators are higher than 0.70, which is higher than the general level. Cronbach's alpha and CR values are higher than 0.8, and the AVE values of all constructs are above 0.5. Therefore, the results of this study show good reliability and convergent validity^38,39^.

**Table 2:** Reliability and convergent validity.

	Indicator	Factor loading	Cronbach’s alpha	CR	AVE
Flow experience	FL1	0.899	0.866	0.918	0.789
	FL2	0.884			
	FL3	0.881			
Platform interactivity	PI1	0.915	0.902	0.939	0.837
	PI2	0.912			
	PI3	0.917			
Participative behaviour	PB1	0.903	0.902	0.919	0.792
	PB2	0.883			
	PB3	0.883			
Sharing behaviour	SB1	0.887	0.897	0.916	0.784
	SB2	0.856			
	SB3	0.913			
Social norms	SN1	0.851	0.875	0.896	0.743
	SN2	0.881			
	SN3	0.853			
Perceived critical mass	CM1	0.825	0.878	0.897	0.744
	CM2	0.887			
	CM3	0.874			

The test of discriminant validity compares the values between AVE and the correlation coefficient of other variables. According to the results in Table 3, the correlation coefficient of the variables is less than the AVE values, which indicates that the discriminant validity of the constructs is also satisfactory^40^.

**Table 3:** AVE values and correlation coefficient of variables.

	PI	FL	CM	SN	PB	SB
PI	0.915					
FL	0.540	0.888				
CM	0.515	0.568	0.863			
SN	0.560	0.601	0.527	0.862		
PB	0.462	0.528	0.508	0.574	0.89	
SB	0.495	0.604	0.615	0.626	0.452	0.885

### Tests of path analysis

Path analysis is a methodological tool for researchers to use quantitative data to explore various causal processes behind specific results. Path analysis is an extension of multiple regression analysis. It estimates the size and intensity of effects in a hypothetical causal system. Because path analysis evaluates the relative strength of different effects on the results, the relationship between variables in the path model is expressed by correlation, so it represents the hypothesis proposed by the researchers^41^.

**Figure 1:**
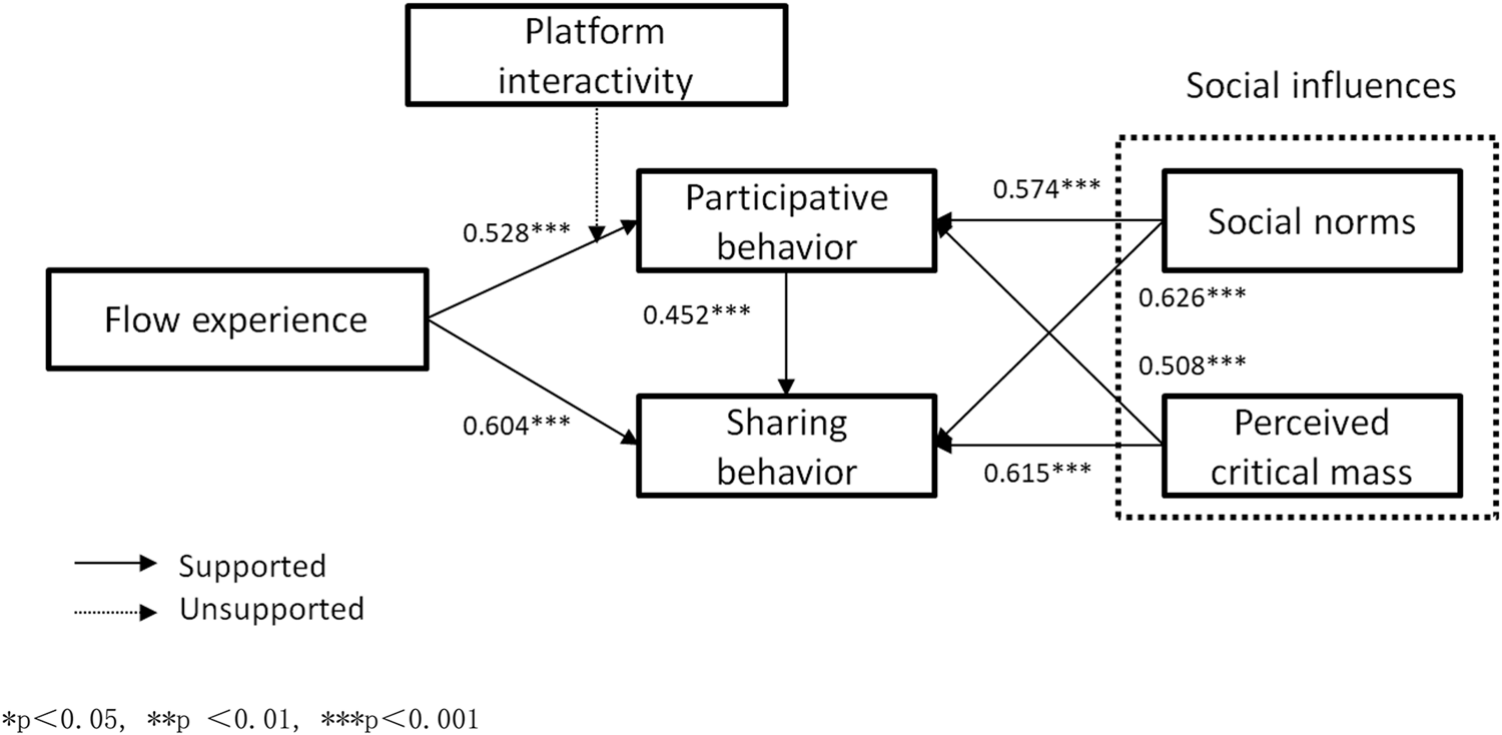
Results of the structural model.

After testing, Table 4 indicates the path coefficient (β), significance (P), t value (t), and R square (R^2^) of the structural model. Participative behaviour in short online videos has a positive significant impact on sharing behaviour (β = 0.615, t = 10.180, P < 0.001, R^2^ = 0.204). Flow experience is positively related to participative behaviour (β = 0.528, t = 12.487, P < 0.001, R^2^ = 0.278) and sharing behaviour. H1, H8 and H9 are supported. Social norms have positive significant effects on participative behaviour (β = 0.574, t = 14.099, P < 0.001, R^2^ = 0.33) and sharing behaviour (β = 0.626, t = 16.137, P < 0.001, R^2^ = 0.392). Finally, perceived critical mass is positively related to participative behaviour (β = 0.508, t = 11.850, P < 0.001, R^2^ = 0.258) and sharing behaviour (β = 0.615, t = 15.647, P < 0.001, R^2^ = 0.378). Therefore, H2 to H5 are supported, as shown in Fig. 1.

**Table 4:** Structural model results.

Structural path	Coefficient	T value	R^2^
FL → PB	0.528***	12.511	0.279
FL → SB	0.604***	15.245	0.365
PB → SB	0.452***	10.180	0.204
SN → PB	0.574***	14.099	0.330
SN → SB	0.626***	16.137	0.392
CM → PB	0.508***	11.850	0.258
CM → SB	0.615***	15.647	0.378

*p < 0.05, **p < 0.01, ***p < 0.001.

### Test of mediation

Mediator variables are behavioural, biological, psychological or sociological structures that transfer the influence of dependent variables to independent variables. Mediation is a way for researchers to explain the process or mechanism by which one variable affects another variable^42^.

**Figure 2:**
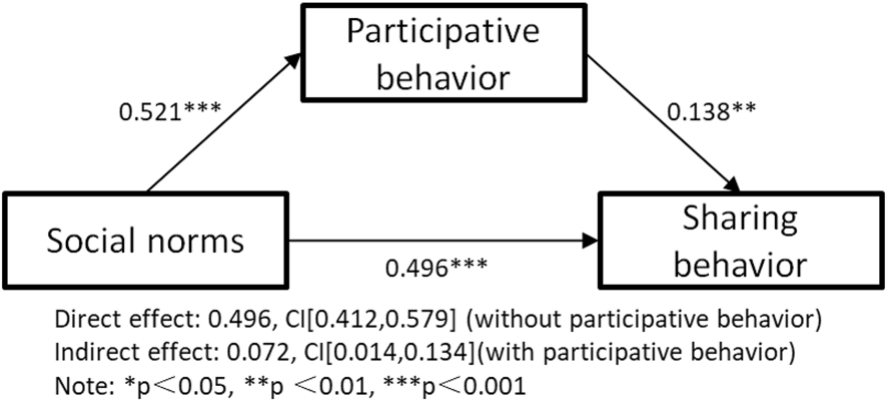
Mediator model testing—Social norms.

H2 to H5, H8 and H9 are valid, illustrating that social norms, perceived critical mass and flow experience, respectively, have a significant impact on the participative behaviour and sharing behaviour of short online videos. Participative behaviour is positively related to sharing behaviour, so the mediating function of participative behaviour between social norms and sharing behaviour is examined by process version 213 in the next test. In this study, the number of bootstrap samples for bias corrected bootstrap confidence intervals is 1000, and the level of confidence for all confidence intervals in the output is 95%. After testing, the direct effect value of social norms on sharing behaviour (without participative behaviour) is 0.496 and the confidence interval (CI) is [0.412, 0.579]. When participative behaviour is tested as a mediator, the indirect effect value of social norms on sharing behaviour is 0.072 and the CI is [0.014, 0.134], excluding 0. Therefore, the mediating function of the participative behaviour of short online video attention between social norms and sharing behaviour is developed and H6 is supported, as shown in Fig. 2.

**Figure 3:**
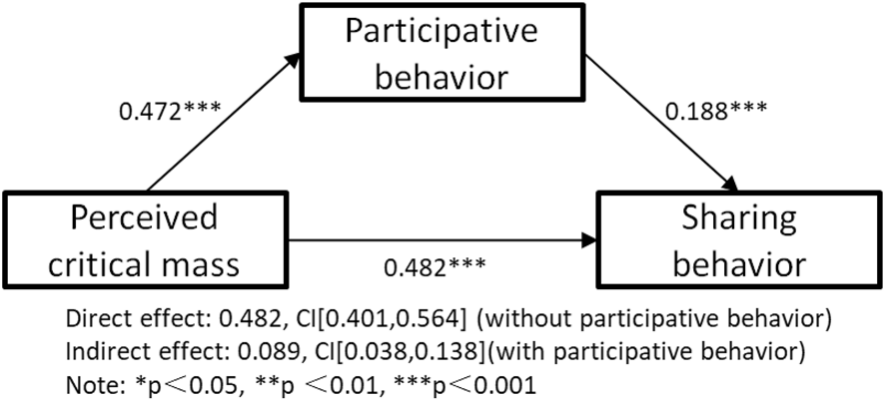
Mediator model testing—Perceived critical mass.

For H7, the direct effect value of perceived critical mass on sharing behaviour (without participative behaviour) is 0.482 and the bootstrap CI is [401, 0.564]. When participative behaviour is tested as a mediating variable, the indirect effect of the perceived critical mass variable on sharing behaviour is 0.089 with CI [0.038, 0.138], excluding 0. Therefore, the mediating role of the participative behaviour of short online videos between perceived critical mass and the sharing behaviour is developed and H7 is supported, as shown in Fig. 3.

**Figure 4:**
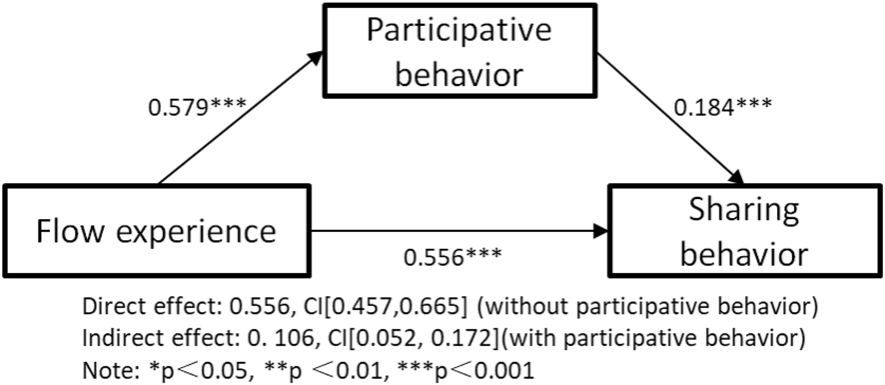
Mediator model testing—flow experience.

For H10, the direct effect value of flow experience on sharing behaviour (without participative behaviour) is 0.556 and the bootstrap CI is [0.457, 0.665]. When participative behaviour is tested as a mediating variable, the indirect effect of the perceived critical mass variable on sharing behaviour is 0.106 with CI [0.052, 0.172], excluding 0. Therefore, the mediating role of the participative behaviour of short online videos between flow experience and sharing behaviour is developed and H10 is supported, as shown in Fig. 4.

### Test of moderation

In statistics, moderating variables mean that the relationship between independent variables and dependent variables changes according to the value of the moderator variable^43^. In addition, moderating variables are essential to assess whether two variables have the same relationship between different groups. In general, the moderating model solves the problem that the variable "when" or "for whom" strongly explains or leads to the result variable^44^.

The final analysis of this study is the moderation of platform interactivity between flow experience and participative behaviour of short online videos by process version 213, with 1000 bootstrap samples for bias corrected bootstrap confidence intervals and a 95% level of confidence for all confidence intervals in the output. After examination, P > 0.05 and the confidence interval is [− 0.124, 0.008], including 0. Accordingly, H11 is unsupported, as shown in Fig. 1.

## Discussion

Among the 10 supported hypotheses, the establishment of H1, H2 and H3 supports Hypothesis H6; H1, H4 and H5 support Hypothesis H7; and H1, H8 and H9 support Hypothesis H10, showing three groups of mediating relationships.

In the first group of mediating relationships, H1, H2, H3, flow experience has a positive significant effect on participative behaviour and sharing behaviour, which shows that flow experience is directly related to user behaviour. Users cannot stop watching short videos at the beginning, and attention is not only focused on video content but can also affect user participation, short video transmission and additional behaviours in this process. It can be seen from the two groups of mediating relationships of social influence that for H1, H4, and H5, social norms have a positive significant effect on participative behaviour and sharing behaviour, which indicates that users' attention to content in a large online user group is not only based on their personal interests but also affected by various social circles. The characteristics of social circles influence individual behaviour and affect users' decisions about video participation and communication. For H1, H8, and H9, perceived critical mass has a positive significant effect on participative and sharing behaviour, indicating that most people in the social circle where users live recognize the participative function of short online videos and form communication behaviour. The exclusive attribute of the social circle further stimulates the short online video platform to generate new users or make user behaviour in the social circle more frequent, thus greatly improving the activity.

For H6, H7 and H10, participative behaviour, as a mediating variable in the three groups of mediation relationships, plays a very important role. In other words, the flow experience, social norms and perceived critical variables in the three mediating groups form video sharing and dissemination through participation. From an overall perspective, this relationship is similar to the concept of an open game. Bernard Suits, an American scholar, believes that an "open game" does not aim to end the game but to keep the game going. He defines an "open game" as a system that can run mutually and whose purpose is to let the game continue^45^. The information participative behaviour formed by praise, collections and comments on short online videos, as a mediating variable, connects the flow experience, social norms and perceived critical variables, forming a circular system of continuous reception, participation and communication. In contrast to network TV, short online videos have a shorter time and higher cycle frequency. Moreover, unlike traditional social media such as text and pictures, video gives users a stronger sense of sensory experience. This open game system is constantly expanding the user group of short online videos, enabling users to be active in the platform and forming continuous viewing and communication behaviour.

As an example of an short online video platform in this research, DouYin is popular. Short online videos have become a new trend in media. This research analyses why people like short videos and why short online videos spread quickly.

In terms of academic implications, this study further expands Novak's research on online flow experience. In particular, previous research on flow experience was often limited to games or online social media based on words and pictures. The relevant exploration of short online videos in this study shows that flow is able to play a role in individual online activities even with the continuous enrichment of sensory experience. At the same time, this study takes the impact of socialization as the measurement variable and confirms that it has a significant positive impact on the behaviour of short online video users. In previous studies when users play online games intensely, interaction with other users leads more people to join^22^. Combined with the results of this study, there are many connections between user behaviour and flow state that forms a kind of open game, promoting short online videos to have a widespread effect in a short time. Therefore, this study also provides a preliminary analysis of the relationship between flow experience and communication.

In terms of social implications, this study confirms that flow experience affects individual behaviour. Network users increasingly expect to enter the flow experience, which makes them lose their sense of time and space^46^ and filters out unnecessary factors^47^. This state caters to people's fragmented life state to some extent^48^, but it may also produce addictive behaviour and affect people's lives, especially for young people. On April 11, 2022, People's Daily published an article on the 10th page reporting that minors are addicted to short video and noting that long-term viewing of short videos will have a serious negative impact on teenagers' physical and mental health. In particular, some teenagers are keen to imitate the characters and actions in short videos, which further affects their healthy growth^49^. According to this study, participative behaviour affects the dissemination of short online videos as a mediating variable between flow experience and social influences. Therefore, when users have addictive behaviour, developers can provide certain network services, weaken the level of function of participative behaviour, and create a healthy network environment for short videos.

In terms of economic implications, participative behaviour and sharing behaviour are indicators of the amount of short videos on the internet. They are related to the flow experience and social influence, which means that users preliminarily identify with the video quality and emotion according to the amount of participative behaviour and sharing behaviour. Research on online games shows that developers should strive to stimulate users' intrinsic motivation as well as their sense of pleasure and satisfaction^50^ and achieve special goals and rewards in execution and confrontation behaviour^51^. Combined with the relevant research on flow experience in the early years, participative behaviour and sharing behaviour of short online videos form a competitive mechanism between producers and sharers that is related to the flow experience and social influence. Therefore, for short online videos, developers could accelerate the network effect and realize the perception of critical groups by participative behaviour and sharing behaviour functions. According to the theory of passive network circulation, the more users view short online videos, the more that users who experience them may communicate to attract other users and produce more services. Finally, developers can obtain more economic reports from users of the virtual community.

## Conclusion and limitations

As a new video medium, short online video has achieved a huge amount of dissemination. It makes up for and occupies the market share of traditional TV, film, text and picture social media. As shown in previous studies^52–57^, flow experience makes users feel happy during network activities. The measurement of social influence in this study also shows that the satisfaction and communication behaviour of short online video users come from their own pleasure and the identity of social groups. Therefore, developers should pay attention not only to the operation mode, reading mode and use habits of the software but also to a series of factors other than software that can stimulate users to produce online social behaviour, such as online community management, user group effects and online social relations, which should be designed as a whole system of open games and concern about their connections.

The results of this study should be treated with caution for the following reasons.

Although the samples are well distributed, some data indicate that the users of DouYin increasingly show a trend of ageing and the development of cities below three or four lines. If a more prominent group is formed, it also presents a direction for the subsequent expansion of the sample range and more accurate locking of the research group. Therefore, for follow-up studies, research on short videos will be based on more models and more variables with reference value for flow experience, short online video watching and other behaviours, providing more comprehensive investigation factors. Finally, this study suggests that researchers of traditional video art should pay attention to the behaviour of audiences with new media and combine it with more personal experience, cultural factors and business behaviour to enrich the research on video art and social media.

## Supplementary Information

Supplementary Information 1.

Supplementary Information 2.

## Data Availability

The datasets used or analysed during the current study are available from the corresponding author on reasonable request.
