# Supplementary material for: Research on the flow experience and social influences of users of short online videos. A case study of DouYin
Source: Sci Rep. 2023 Feb 27;13:3312. doi: 10.1038/s41598-023-30525-y (PMC9970967; doi:10.1038/s41598-023-30525-y)
Supplement: Supplementary file 1 — Supplementary Information 1. [file 41598_2023_30525_MOESM1_ESM.doc]

Research on low experience and social influences of short video.

A case study of DouYin.

Cheng Zheng

Appendix:

| Variables | References | items |
| --- | --- | --- |
| Flow experience（FL） | (Chang,2013[[1]](#endnote-2)) | FL1: When I was watching short online videos, I felt totally captivated.  FL2: When I was navigating short online videos, time seemed to pass very quickly.  FL3: It is hard to control how much time I spend when browsing short online videos. |
| Platform interactivity(PI) | (Chang,2013Error: Reference source not found;Lee, 2009[[2]](#endnote-3)) | PI1: A mainstream short online video platform can provide a good multimedia user interface.  PI2: A mainstream short online video platform can provide a good navigation structure. I can always find the video I want and the author I follow.  PI3: A mainstream short online video platform can provide a good visual system that is easy to watch. |
| Participative behaviour（PB） | (Chen Yu & Wang Xindi ,2020[[3]](#endnote-4)) | PB1: I often make comments, praise and collection decisions for DouYin online video content or authors. PB2: When I use DouYin to watch short online videos frequently, comments are often made. PB3: In the process of using DouYin to watch short online videos, people often praise or collect them. |
| Sharing behaviour (SB) | (Chen Yu & Wang Xindi ,2020Error: Reference source not found) | SB1: When i using DouYin, i often create my own short online videos. SB2: In the process of using DouYin, I often forward short online videos created by others. SB3: During the use of DouYin, I often forward short online videos of my own creation. |
| Social norms(SN) | (Hsu C L, Lu H P,2004[[4]](#endnote-5)) | SN1: My family members think that I should play DouYin for short online videos.  SN2: My classmates think that I should play DouYin for short online videos.  SN3: My friends think that I should play DouYin for short online videos. |
| Perceived critical mass(CM) | (Hsu C L, Lu H P,2004Error: Reference source not found) | CM1: Most people in my group play DouYin for short online videos frequently. CM2: Most people in my community play DouYin for short online videos frequently.  CM3: Most people in my class/office play DouYin for short online videos frequently. |

1. Chang, C.-C. Examining users′ intention to continue using social network games: A flow experience perspective. Telematics and Informatics 30, 311-321 (2013). [↑](#endnote-ref-2)
2. Lee, M. C. Understanding the behavioural intention to play online games: An extension of the theory of planned behaviour. Online information review (2009). [↑](#endnote-ref-3)
3. Chen, Y. & Wang, X. D. A study on the continuous use behavior of weibo users’ immersive experience in the new media era: Effect of potential affordability regulation. Library 63–71 (2020). [↑](#endnote-ref-4)
4. Hsu, C.-L. & Lu, H.-P. Why do people play on-line games? An extended TAM with social influences and flow experience. Information & management 41, 853-868 (2004). [↑](#endnote-ref-5)
