# Supplementary material for: Research on the flow experience and social influences of users of short online videos. A case study of DouYin
Source: Sci Rep. 2023 Feb 27;13:3312. doi: 10.1038/s41598-023-30525-y (PMC9970967; doi:10.1038/s41598-023-30525-y)
Supplement: Supplementary file 2 — Supplementary Information 2. [file 41598_2023_30525_MOESM2_ESM.pdf]

Research on the flow experience and social influences of users of short online videos.
A case study of DouYin.

Cheng Zheng Ph.D.

totalseconds	Age	Sex	Education	City	PI1	PI2	PI3	FL1	FL2	FL3	PB1	PB2	PB3	SB1	SB2	SB3	SN1	SN2	SN3	CM1	CM2	CM3
1	2	1	2	2	4	4	4	3	3	4	4	5	4	4	4	5	4	4	5	4	4	5
2	1	2	2	3	2	3	2	3	4	4	4	3	3	4	5	4	3	3	3	2	2	2
3	2	2	2	4	4	4	4	3	3	2	5	4	4	4	5	4	1	3	4	5	2	4
4	2	2	1	4	7	4	7	7	5	5	6	4	7	7	5	6	5	7	6	6	7	5
5	4	2	3	2	2	2	1	7	7	5	6	7	7	4	7	7	4	7	7	7	5	7
6	4	2	2	3	4	4	4	3	3	3	2	2	3	3	3	2	2	2	2	3	3	1
7	2	2	2	2	2	2	2	4	5	5	4	5	4	4	3	4	3	1	1	4	3	3
8	2	1	2	2	2	3	3	3	3	3	4	4	5	4	4	4	4	2	1	4	2	3
9	2	2	2	3	5	4	4	5	4	4	4	4	4	4	5	5	5	5	5	3	3	3
10	2	2	4	2	5	5	6	7	6	5	6	7	4	5	7	6	5	6	7	7	5	7
11	3	1	2	4	5	4	6	6	4	5	3	3	3	6	6	7	4	6	6	7	7	4
12	2	2	2	1	4	4	4	4	5	5	4	4	4	5	4	5	5	3	5	4	4	5
13	2	1	2	2	2	2	2	2	4	3	7	7	7	5	4	5	2	3	1	2	2	2
14	3	2	4	1	4	5	3	5	4	4	5	4	4	3	5	4	2	3	2	5	3	4
15	2	2	2	4	7	6	7	5	5	6	5	4	7	5	7	4	5	7	7	5	5	7
16	4	2	2	3	3	2	3	2	4	2	4	5	5	2	2	3	1	5	5	2	2	1
17	2	2	2	3	5	4	5	7	4	7	4	5	5	4	5	5	5	5	5	5	4	4
18	4	1	4	1	4	4	4	3	5	5	4	4	5	4	5	3	4	3	3	3	3	3
19	2	2	1	3	1	1	1	3	3	3	3	5	3	5	4	4	4	4	4	3	5	2
20	2	1	2	1	6	4	7	7	7	6	6	5	7	6	7	7	6	7	6	6	5	5
21	3	2	3	4	7	5	7	7	5	6	6	6	5	7	5	5	6	5	7	7	5	5
22	2	2	2	3	4	4	3	5	5	4	4	4	5	4	4	4	4	4	4	3	3	4
23	3	1	2	1	4	5	5	4	4	3	4	4	4	5	4	5	4	4	4	3	4	5
24	2	1	2	3	2	2	3	5	5	5	4	5	4	5	4	5	2	5	5	3	3	4
25	4	2	4	1	5	4	5	3	3	4	4	5	5	5	4	4	3	3	3	4	2	5
26	3	1	3	3	7	7	4	7	7	7	3	3	3	7	6	7	6	7	5	3	4	3
28	1	2	2	4	4	5	5	5	5	5	4	4	4	4	5	5	5	1	1	5	5	5
29	4	1	1	4	7	7	7	7	7	7	6	7	7	6	7	5	1	3	2	4	3	4
30	1	2	2	3	7	5	5	6	7	5	4	7	7	7	7	7	6	6	5	5	5	7
31	2	2	2	2	1	1	2	3	4	3	5	4	4	4	4	5	3	4	5	4	4	4
32	2	2	3	2	5	4	5	5	5	4	4	5	5	4	4	4	5	5	5	2	2	2
33	2	1	1	1	4	5	5	5	4	4	5	5	4	1	2	1	5	5	5	1	1	1
34	2	1	3	4	4	4	5	4	4	5	5	5	5	5	5	3	5	3	4	3	4	4
35	2	2	3	1	3	3	3	3	3	3	3	3	3	3	3	3	3	3	3	3	3	3
36	3	1	3	3	4	5	4	6	5	6	4	4	5	5	5	4	5	4	4	2	5	5
37	4	1	1	4	4	5	4	4	4	3	4	3	4	4	4	5	3	5	1	5	4	4
38	3	2	3	4	5	4	5	5	4	4	4	5	4	5	5	4	5	5	5	4	3	3
39	3	2	4	2	4	5	5	3	5	5	3	5	4	4	4	4	4	4	5	3	4	4
40	4	2	2	1	4	5	5	1	5	3	3	2	3	3	2	3	3	2	2	2	2	2
41	2	2	2	2	4	3	4	5	4	3	5	5	5	4	5	4	3	3	3	5	5	5
42	3	1	2	4	6	7	7	2	4	2	4	7	4	5	5	7	7	7	7	5	7	7
43	2	2	3	3	5	7	7	5	5	5	2	4	3	7	6	7	5	7	5	7	4	7
44	3	2	4	1	5	3	4	5	5	5	4	5	4	5	4	5	3	5	5	3	5	5
45	2	2	3	4	5	4	4	3	3	2	4	5	5	2	2	2	2	3	3	1	3	4
46	3	1	2	3	7	7	6	7	6	6	7	6	5	5	5	6	4	5	5	6	7	7
47	4	1	4	3	5	5	7	5	7	7	3	4	2	7	7	6	5	6	4	6	6	7
48	2	2	2	2	5	7	7	7	5	7	6	4	7	7	6	7	5	6	7	6	5	5
49	4	1	2	3	5	4	4	2	3	4	5	3	4	1	2	2	1	1	5	3	3	1
50	2	1	3	3	7	6	5	5	7	7	5	5	7	7	7	5	7	6	7	4	3	2
51	4	2	1	4	4	4	4	5	5	5	4	5	4	1	1	2	3	5	4	1	2	3
52	4	2	2	4	4	7	5	5	5	7	7	7	7	5	6	4	7	5	5	6	5	6
53	4	1	2	4	6	5	4	5	5	5	7	5	7	7	6	4	2	4	3	4	6	7
54	2	2	2	4	4	5	5	4	4	4	4	4	5	5	5	3	3	2	3	4	4	5
55	2	2	2	2	5	4	4	5	5	5	4	5	4	4	3	4	3	5	5	5	2	2
56	4	2	1	2	5	4	5	5	4	5	4	4	4	4	5	5	3	5	4	2	3	4
57	2	1	2	1	4	4	4	5	4	4	4	5	5	4	5	5	4	4	4	5	4	5
58	2	1	3	2	3	3	3	4	4	4	3	3	3	3	3	3	4	4	4	4	4	3
59	4	2	3	4	5	4	5	5	5	5	5	4	4	5	5	5	5	5	5	5	5	3
60	4	1	2	2	3	4	4	4	5	4	5	5	4	1	1	1	2	2	4	4	4	4
61	2	2	3	3	3	2	3	4	4	5	1	3	2	3	4	4	2	4	5	1	1	2
62	2	2	2	3	5	3	4	4	4	4	5	5	5	4	4	4	5	5	5	3	3	3
63	4	1	1	3	4	5	5	4	2	4	5	4	4	4	4	4	4	4	4	3	3	4
64	2	2	2	2	6	7	5	7	6	5	7	7	7	4	7	4	4	7	5	6	7	5
65	3	2	2	1	5	5	5	5	5	7	4	5	5	5	4	5	5	5	5	5	5	4
66	4	1	1	4	7	7	6	7	6	5	6	6	7	5	4	7	7	7	5	6	4	5
67	2	1	2	3	4	5	4	6	6	6	4	5	4	4	5	5	5	5	5	4	5	5
68	4	2	2	1	6	5	6	7	6	7	6	6	5	4	7	7	6	7	5	6	6	7
69	4	1	2	2	4	4	4	4	4	5	4	5	5	1	3	1	2	2	2	5	5	3
70	2	2	3	3	5	7	4	6	6	5	5	6	7	3	2	2	5	5	6	4	7	7
71	2	1	3	3	7	5	5	7	6	5	7	6	4	5	7	5	6	7	6	6	7	5

72	2	2	4	1	5	6	7	7	5	7	5	5	6	7	6	6	6	4	7	1	1	2
73	2	2	1	3	6	5	5	5	5	6	5	6	7	3	3	3	5	6	7	5	5	5
74	4	1	2	3	4	5	5	5	5	5	4	4	5	5	5	3	3	3	3	4	4	5
75	3	2	2	3	4	5	5	4	5	3	4	5	4	4	5	4	4	4	4	3	4	3
76	3	1	2	2	3	4	4	5	5	4	4	5	4	5	5	5	5	5	5	3	3	2
77	3	2	1	1	4	5	3	4	4	4	4	4	5	4	4	3	1	4	3	4	4	4
78	3	2	2	1	5	5	5	4	4	6	5	5	4	4	5	4	5	5	5	5	3	2
79	2	1	2	3	7	6	7	5	6	6	6	5	7	5	6	5	7	7	7	6	7	7
80	4	2	1	1	5	4	4	4	5	5	4	5	4	5	3	5	3	4	5	3	4	5
81	2	1	2	3	6	6	6	7	7	5	5	7	5	6	5	7	7	7	5	5	7	7
82	4	2	2	2	3	5	5	5	4	5	5	3	4	4	4	5	4	4	2	5	5	5
83	2	2	2	2	3	2	3	5	5	5	5	3	4	5	4	5	5	3	4	3	4	4
84	4	2	2	3	5	7	7	6	6	7	2	2	2	6	6	5	4	7	5	5	6	7
85	3	2	2	3	5	5	5	5	3	5	4	5	5	4	5	4	4	4	1	5	3	4
86	2	2	2	4	5	5	3	2	3	4	2	2	3	3	3	3	1	1	5	3	3	1
87	2	2	2	1	4	3	5	5	5	4	4	4	4	4	4	4	3	5	5	3	2	4
88	3	1	3	2	4	5	5	3	2	3	4	4	5	3	1	2	5	5	1	2	2	2
89	2	1	3	3	1	3	2	2	4	3	2	2	2	2	2	2	1	1	1	2	2	2
90	1	1	1	3	4	3	5	3	4	3	3	3	3	2	2	2	1	1	5	1	3	3
91	4	2	3	2	3	1	2	3	3	3	3	2	2	2	2	2	2	2	2	2	2	2
94	2	1	2	1	5	3	4	5	5	5	5	4	5	3	4	4	3	4	4	3	4	4
95	4	2	2	2	5	5	3	5	5	4	4	4	5	5	4	5	4	5	5	4	2	4
96	2	2	2	3	7	7	7	7	5	7	6	6	5	6	6	6	6	6	7	7	6	5
97	4	1	2	2	2	1	2	3	4	4	2	1	2	5	4	5	3	3	3	3	3	5
98	2	2	2	3	1	3	2	2	4	4	1	1	3	5	5	5	2	1	1	1	5	4
99	1	2	1	1	3	5	5	3	3	3	4	4	4	1	2	1	2	3	2	2	2	2
100	1	2	2	2	1	1	2	2	2	2	1	1	3	2	2	2	3	3	3	1	1	1
101	4	1	3	4	4	4	4	6	6	7	5	5	5	5	4	5	5	5	5	5	5	5
102	2	1	3	4	7	5	5	5	5	6	5	7	6	6	5	6	7	5	4	4	7	5
103	2	2	2	4	2	3	1	4	3	1	3	2	2	2	2	2	2	3	1	2	2	2
104	2	2	2	4	5	5	5	5	5	5	3	3	2	4	5	5	5	5	5	2	2	2
105	4	2	2	2	3	5	5	5	5	4	4	5	4	5	4	5	5	5	5	3	3	3
106	2	1	3	2	5	5	5	5	6	5	5	5	5	5	5	5	5	5	5	5	5	5
107	2	2	1	2	4	3	5	4	5	5	5	5	5	5	4	4	5	5	3	4	4	4
108	4	2	3	3	4	4	4	3	3	3	5	4	4	3	5	5	5	5	5	1	1	1
109	1	2	1	2	2	2	1	3	2	3	2	3	4	4	4	3	5	5	5	5	5	5
110	3	2	2	4	7	5	7	5	5	7	6	4	7	7	6	6	6	5	5	2	3	4
111	2	2	2	2	1	2	1	3	4	4	4	4	5	3	5	4	3	3	3	4	3	3
112	3	1	3	1	5	4	3	2	3	4	4	4	4	4	4	5	1	4	4	5	3	4
113	2	2	2	2	5	4	5	4	3	1	5	5	5	4	5	4	5	5	5	5	4	5
114	1	1	1	3	5	6	5	5	7	6	5	6	5	6	6	7	7	5	5	4	1	1
115	1	1	2	2	2	3	2	2	3	4	4	5	5	1	3	2	3	2	2	2	2	2
116	4	1	4	1	7	6	6	5	7	5	5	6	4	7	7	7	7	5	6	7	5	7
117	4	2	2	2	6	7	6	6	7	5	3	3	3	7	5	7	7	4	5	7	7	6
118	3	2	3	2	5	4	3	4	3	4	4	4	5	4	4	4	3	3	5	4	4	5
119	4	1	3	2	4	5	5	4	4	3	4	4	4	3	2	1	1	1	1	1	1	5
120	3	2	2	3	4	4	3	3	4	3	2	3	3	3	4	3	4	4	3	4	4	3
121	2	1	2	4	2	2	3	4	4	5	4	4	4	3	4	4	3	5	1	5	4	4
122	2	2	2	3	7	6	5	5	6	6	1	1	4	5	7	7	6	6	6	7	6	7
123	3	2	1	3	5	4	4	3	3	5	5	4	5	5	4	4	5	5	5	3	2	4
124	4	2	2	2	7	7	4	7	7	4	7	6	6	7	5	6	6	6	7	5	7	7
125	2	1	3	3	6	6	7	6	6	5	5	5	5	5	5	5	7	5	5	5	6	5
126	4	2	4	2	2	2	3	3	3	3	2	2	2	2	4	4	4	4	3	3	3	3
127	1	2	1	2	1	2	2	4	4	4	2	2	2	5	4	5	1	5	5	4	4	4
128	2	1	2	3	4	4	3	5	5	5	5	4	4	4	4	5	3	5	3	4	4	5
129	2	2	2	2	7	7	5	1	3	4	7	7	4	5	6	6	7	6	7	7	4	5
130	2	1	4	2	4	5	5	1	5	2	1	1	2	2	2	1	2	2	2	2	2	2
131	4	1	2	2	5	5	4	5	7	6	4	5	4	5	5	5	5	5	5	4	4	4
132	2	1	2	2	5	4	5	5	3	2	1	3	1	5	4	5	3	3	3	3	3	5
133	4	1	3	2	4	5	5	5	5	5	4	4	5	4	5	3	5	4	4	4	4	5
134	2	1	3	2	4	5	4	4	4	4	4	4	4	3	3	3	1	5	3	1	3	3
135	4	2	3	4	5	5	4	5	6	6	5	5	4	5	5	5	5	5	5	5	5	5
136	4	2	2	3	5	5	4	5	5	4	2	2	2	4	5	5	2	3	2	5	5	4
137	3	1	2	3	4	5	5	4	5	4	5	5	3	4	5	4	3	3	2	3	3	5
138	4	2	1	3	4	4	3	5	5	5	4	5	4	5	4	5	4	4	4	3	5	4
139	3	2	4	2	4	5	3	3	2	3	5	5	5	2	2	1	3	3	3	3	3	4
140	4	1	2	4	3	3	3	5	6	7	6	7	6	5	6	5	6	6	6	7	6	7
141	3	1	4	1	7	7	4	6	7	5	4	5	6	5	7	7	6	4	6	7	7	6
142	2	2	1	3	2	3	3	3	3	4	4	4	5	1	3	2	2	2	2	2	3	2
143	4	1	4	1	5	5	4	3	3	4	4	4	5	4	5	4	3	4	5	4	4	5
144	2	1	2	3	2	4	3	7	7	5	4	5	6	6	6	7	4	6	5	5	7	4
145	2	2	2	3	4	3	4	3	3	3	5	4	5	4	5	5	3	3	2	3	3	4
146	2	2	3	4	4	5	3	2	3	3	2	3	3	2	2	3	3	3	3	1	1	1
147	4	2	2	3	5	4	5	3	2	2	4	4	4	5	3	4	4	5	5	4	2	4
148	1	1	1	3	7	6	5	6	6	7	6	4	4	5	6	5	7	5	5	7	5	7
149	2	1	2	2	1	3	2	4	4	4	5	4	5	5	3	5	3	3	5	1	3	3

150	2	2	2	4	4	7	7	5	5	6	5	4	5	7	6	5	4	7	5	7	7	5
151	2	1	2	3	1	1	1	4	5	5	3	4	4	4	4	5	2	3	3	4	4	4
152	4	1	3	3	1	1	2	3	4	5	2	2	3	5	5	4	5	1	5	4	4	4
153	4	1	1	4	5	5	4	6	6	5	4	5	4	4	5	5	5	5	5	3	3	5
154	4	1	3	3	6	5	6	6	7	7	5	5	5	6	6	5	6	5	6	1	4	3
155	1	2	2	2	6	5	5	5	5	7	7	5	7	7	6	6	7	5	7	3	2	3
156	3	1	3	3	5	6	7	4	7	7	6	6	6	6	7	7	5	5	4	6	7	6
157	4	1	3	3	2	3	1	4	4	4	2	3	2	5	4	4	3	3	3	3	3	3
158	2	2	2	2	4	4	5	4	5	4	2	2	3	3	5	4	1	2	4	4	4	5
159	2	2	2	3	4	3	5	3	2	3	3	2	3	3	2	3	3	1	1	2	3	1
160	4	2	2	2	5	5	7	5	4	6	7	7	7	5	7	6	4	6	7	5	6	4
161	1	2	2	2	7	4	4	5	6	6	6	5	7	7	4	7	5	5	7	5	5	6
162	3	1	2	2	4	5	4	5	5	5	5	4	5	4	4	3	3	3	3	4	3	5
163	1	1	2	3	1	2	2	4	4	5	4	5	4	1	3	2	2	3	3	4	4	5
164	3	2	2	3	3	3	3	3	3	3	3	3	3	3	3	3	3	3	3	3	3	3
165	2	2	2	3	2	2	2	3	1	5	3	3	2	2	3	2	3	2	2	2	2	2
166	4	1	2	3	5	4	4	4	4	4	1	1	3	4	4	4	2	1	1	3	3	3
167	2	2	2	4	4	4	5	5	5	5	4	4	4	5	5	4	1	1	1	4	4	5
168	4	1	2	3	5	5	5	5	4	4	1	1	3	5	4	4	2	1	2	4	3	4
169	4	1	2	2	1	2	2	3	3	3	5	4	5	5	4	5	3	2	5	2	4	4
170	4	2	2	4	4	5	5	7	7	6	5	5	5	5	4	3	5	5	5	5	5	5
171	4	1	2	3	5	7	7	4	2	3	6	6	6	4	3	2	5	7	6	7	7	7
172	4	1	2	3	4	4	4	3	4	3	2	2	3	5	5	4	1	4	1	5	5	5
173	4	2	2	2	2	3	1	1	1	1	1	3	1	1	1	1	1	1	1	1	3	1
174	4	2	2	2	5	5	7	7	6	7	7	7	5	5	7	5	6	7	6	5	6	6
175	4	1	2	4	5	4	4	3	3	3	4	5	5	1	1	1	2	2	2	4	5	5
177	4	2	3	3	4	3	1	4	5	5	7	7	4	4	7	7	4	5	5	6	6	7
178	2	1	2	3	4	5	5	5	5	4	5	4	4	2	1	2	3	3	3	3	3	3
179	2	1	3	2	4	5	5	5	5	5	5	5	3	4	4	4	3	3	5	1	3	3
180	1	1	1	3	5	4	4	4	6	5	5	5	4	5	4	4	5	4	5	5	4	5
181	2	2	2	3	3	4	4	5	5	5	4	3	4	5	4	4	1	4	1	4	4	4
182	4	2	4	1	3	5	5	4	5	5	5	4	4	5	4	3	3	5	5	4	2	5
183	4	2	1	3	4	5	4	3	3	4	2	3	3	2	2	2	2	2	2	2	2	2
184	4	2	3	4	7	7	7	7	5	5	6	5	7	5	6	7	6	6	5	5	7	5
185	2	2	2	3	7	7	7	7	7	6	7	7	5	7	7	4	7	5	5	6	7	6
186	4	2	2	2	5	7	7	4	5	5	5	5	5	7	5	6	4	3	4	7	4	5
187	2	2	2	2	4	4	4	1	5	3	4	5	5	1	2	1	1	1	1	2	2	2
188	3	1	3	3	7	5	6	4	5	6	5	7	6	5	7	7	7	7	7	6	7	5
189	4	1	4	1	1	3	2	3	3	4	3	2	3	2	3	2	3	3	3	1	1	1
190	3	2	2	3	3	2	2	3	3	3	4	3	3	3	4	4	5	5	4	3	3	3
191	4	2	2	2	2	2	1	4	4	3	4	4	4	5	3	5	2	3	3	1	3	4
192	2	2	2	3	7	6	7	5	7	6	6	4	5	6	5	7	7	6	6	5	5	5
193	1	1	2	2	5	4	5	5	5	5	5	5	4	4	5	3	3	4	3	2	4	5
194	4	2	2	2	6	5	5	6	5	4	7	6	7	5	6	7	6	6	5	3	3	3
195	2	2	2	2	2	3	3	4	4	5	5	5	4	3	1	1	1	1	1	1	2	2
196	2	1	2	3	5	4	4	3	3	4	2	2	2	5	4	5	2	2	2	2	3	2
197	4	1	2	3	5	4	5	3	3	4	3	3	3	3	3	2	2	2	2	2	3	2
198	1	2	2	3	3	5	4	3	3	3	2	1	2	5	5	5	2	2	2	3	3	5
199	2	2	2	3	5	4	4	5	5	4	5	5	5	4	5	5	4	4	5	3	4	4
200	3	2	2	3	4	7	5	6	4	4	4	4	7	7	6	7	5	6	7	6	7	6
201	2	2	2	4	5	5	3	3	3	3	4	5	4	4	5	5	5	5	5	1	1	1
202	1	2	2	3	2	3	3	2	4	3	2	2	2	1	2	2	3	2	2	2	2	2
203	4	1	4	1	4	5	4	4	5	5	5	5	3	5	4	5	3	3	5	3	4	4
204	2	2	2	2	2	2	1	7	4	6	7	7	7	6	6	5	7	5	5	6	6	6
205	4	2	2	3	5	5	4	1	2	2	5	4	5	5	4	5	5	5	5	4	4	4
206	3	2	2	3	4	4	5	4	5	4	1	2	2	5	4	4	2	2	2	4	4	4
207	4	2	4	2	4	3	4	5	6	5	4	5	4	4	5	5	5	5	5	5	3	2
208	4	1	2	3	5	4	5	4	4	4	4	4	5	5	5	5	5	5	5	2	2	2
209	1	2	2	2	4	5	5	3	3	3	3	2	2	2	3	3	4	4	3	3	3	3
210	2	2	3	3	3	4	4	4	4	4	4	4	4	4	3	5	3	2	5	2	4	4
211	3	2	2	2	1	1	1	1	1	1	1	1	1	1	2	1	1	1	1	1	1	4
212	2	2	2	2	2	2	2	4	4	4	2	1	1	4	5	5	2	2	1	4	3	3
213	1	1	2	3	7	6	7	7	6	6	3	1	3	5	7	5	7	7	5	5	4	5
214	2	1	2	3	3	4	4	5	5	5	5	4	5	4	4	5	3	3	3	5	5	5
215	2	2	2	4	3	5	4	3	3	3	4	4	5	2	1	2	2	4	3	1	1	1
216	4	1	2	2	5	4	5	4	5	5	4	4	4	5	3	5	5	5	5	2	2	2
218	2	1	2	4	5	4	5	3	3	3	4	5	4	4	5	4	3	3	3	4	4	5
220	2	1	2	3	3	2	3	4	3	4	3	2	3	2	3	2	3	2	2	2	2	3
221	3	1	4	1	5	5	4	3	3	3	4	5	4	5	4	3	3	5	5	2	2	5
222	4	2	2	2	4	4	5	2	3	2	4	3	4	5	5	3	3	3	3	4	4	4
223	4	2	2	2	5	4	4	5	5	5	4	5	4	5	5	3	3	3	3	2	3	4
224	3	1	2	2	7	6	7	5	6	5	7	7	6	6	6	5	7	5	7	3	3	4
225	4	1	2	3	4	5	5	1	4	2	5	5	4	5	4	5	5	5	5	4	4	4
226	1	2	2	2	3	4	2	7	7	6	6	5	6	7	4	5	3	4	4	7	6	7
227	4	2	2	3	4	7	5	7	6	5	7	7	7	6	7	7	6	7	5	7	5	5
228	4	1	3	2	4	4	5	3	2	3	1	1	2	1	3	2	3	3	3	2	2	2

229	3	1	2	4	4	4	4	5	5	5	5	5	5	4	3	4	4	4	4	4	4	4
230	4	1	2	3	5	4	5	5	4	4	4	4	4	5	4	5	5	3	5	4	4	5
231	2	1	4	1	7	6	5	5	6	5	4	7	6	6	6	5	5	6	5	6	5	7
232	1	2	1	4	4	5	5	5	5	5	3	5	4	5	5	5	3	3	3	4	4	3
233	2	2	2	2	5	5	5	5	5	5	5	3	4	5	5	5	3	4	5	4	4	4
234	2	2	2	3	3	2	3	5	5	5	4	4	5	4	5	4	3	3	3	5	5	3
235	3	1	4	2	4	4	5	3	3	4	2	1	1	5	4	5	1	4	4	4	4	3
236	4	1	4	1	3	3	2	3	2	3	4	3	4	3	5	4	1	2	3	3	2	4
237	4	2	2	2	4	4	5	4	5	5	5	5	5	4	3	5	5	5	5	4	4	4
238	2	1	2	3	5	4	4	3	3	4	4	5	5	4	5	4	4	4	5	4	4	5
239	2	1	3	3	2	1	2	2	3	4	5	4	4	4	4	4	3	3	4	2	3	3
240	3	2	2	2	5	7	4	6	6	7	6	6	7	5	6	6	6	7	6	7	5	5
241	4	1	2	3	1	1	1	3	2	4	5	4	4	1	3	2	2	3	2	2	2	2
242	2	2	2	2	3	2	2	3	3	3	1	2	2	2	2	2	2	3	2	2	2	2
243	4	2	2	2	3	5	4	4	3	3	5	5	4	3	4	4	4	4	4	3	3	3
244	2	2	3	2	5	4	5	6	5	6	5	4	5	4	5	5	5	1	3	5	5	5
245	3	1	2	4	5	7	5	6	7	4	2	1	3	6	6	5	3	3	3	7	6	6
246	4	1	2	3	5	5	4	7	7	7	7	5	5	5	7	7	5	7	5	2	3	4
248	2	1	4	1	3	5	4	5	4	4	2	2	2	5	4	5	3	3	2	4	4	4
249	2	2	2	3	4	7	6	6	6	6	7	5	7	7	6	6	4	6	7	5	6	7
250	3	1	2	3	5	5	4	4	4	4	5	4	4	5	3	4	5	5	5	5	1	1
251	2	1	2	2	7	7	6	5	6	6	6	7	7	6	7	7	2	1	4	7	5	7
252	1	2	2	4	3	3	3	3	3	3	5	4	4	5	4	5	3	3	3	4	3	5
253	2	2	2	3	4	3	4	3	3	3	3	4	3	5	4	4	4	4	4	3	4	5
254	2	1	2	3	7	7	5	6	6	7	5	6	7	5	7	5	7	7	7	7	4	6
255	4	2	2	4	2	2	1	3	3	3	5	4	4	3	5	4	1	3	5	5	4	4
256	2	2	2	2	6	6	7	5	4	7	6	6	5	6	5	6	7	6	6	7	4	6
257	4	2	3	2	4	5	4	4	4	4	2	2	2	4	4	4	2	3	4	2	3	3
259	3	1	1	3	3	5	4	4	5	4	4	5	4	4	5	5	1	1	1	4	4	4
260	3	2	2	4	4	5	5	4	4	3	5	4	4	4	5	5	5	1	5	4	4	4
261	1	1	1	4	5	4	5	5	3	3	2	2	2	4	4	4	3	3	4	2	3	4
262	2	1	2	2	7	5	6	7	5	6	3	3	3	6	4	6	7	5	7	5	7	7
263	2	2	3	4	5	5	5	7	4	7	7	7	5	4	6	5	6	4	7	6	6	5
264	2	2	1	3	5	5	7	7	7	7	5	6	5	4	3	4	5	5	7	5	6	6
265	3	1	2	3	5	4	5	5	5	5	5	4	5	1	2	1	4	4	4	1	1	5
266	2	1	2	2	7	7	5	6	5	5	7	7	6	7	6	5	6	6	4	7	7	4
267	4	1	2	4	7	6	5	6	7	5	5	7	6	4	5	7	5	6	6	7	7	6
268	2	2	2	3	5	5	3	3	3	1	3	3	3	3	2	3	3	1	2	1	5	4
269	3	2	2	3	5	4	4	3	3	3	5	5	4	3	2	3	4	4	3	5	2	3
270	2	2	2	3	1	1	1	3	3	4	1	1	3	5	5	5	5	5	5	1	1	1
271	4	1	2	3	4	2	3	5	6	7	7	7	7	4	6	7	6	7	6	5	5	5
272	2	1	2	3	4	5	5	4	4	5	4	5	4	3	4	4	2	4	4	4	4	4
273	2	2	3	3	4	6	6	3	4	2	7	5	6	6	7	5	5	5	7	7	7	7
274	2	1	3	3	6	6	6	4	5	7	7	4	6	5	6	6	5	5	5	6	6	7
275	2	2	2	3	6	4	6	5	7	6	5	7	5	5	5	7	7	6	7	6	6	7
276	2	2	2	4	4	5	5	4	3	4	5	4	4	5	5	4	5	5	5	2	2	2
277	2	1	4	1	4	3	4	3	3	3	2	3	2	2	3	2	1	1	1	1	2	2
278	4	2	4	3	4	5	5	3	4	4	4	4	4	4	5	4	5	5	5	1	1	1
279	4	1	2	3	4	4	5	4	3	4	5	5	3	4	5	5	4	5	3	3	3	3
280	2	2	2	4	4	5	3	5	5	5	4	3	5	5	5	4	4	4	2	4	4	4
281	3	1	2	2	5	3	5	5	5	5	4	4	4	5	4	5	4	5	5	4	4	4
282	1	1	1	2	5	5	5	6	7	5	5	5	7	6	5	7	4	6	5	5	5	7
283	4	1	2	3	5	4	4	4	2	3	2	2	2	3	2	2	2	2	2	2	3	2
284	4	2	2	2	4	4	4	3	4	4	2	2	1	5	5	4	4	2	3	3	4	5
285	4	1	2	3	5	4	3	4	3	5	5	5	4	4	4	4	3	4	4	4	3	5
286	2	1	1	4	4	4	4	3	3	3	2	1	2	4	4	4	4	1	1	3	5	4
287	4	1	2	2	5	4	4	5	5	5	4	5	4	5	4	5	5	5	3	5	3	4
288	4	2	2	3	4	5	4	4	4	4	5	5	5	5	4	4	2	3	3	4	4	5
289	2	1	2	2	5	5	4	4	6	6	4	5	5	5	5	5	5	5	5	5	5	5
290	3	1	1	2	2	2	1	3	3	4	3	2	3	3	2	3	2	4	3	1	1	1
291	4	2	4	1	4	5	4	3	3	3	2	2	1	1	2	2	1	1	1	2	2	2
292	2	2	2	2	3	2	1	5	5	5	4	3	4	5	5	5	3	3	3	3	3	5
293	2	1	2	3	4	5	5	4	5	4	5	5	5	5	5	3	1	1	4	4	4	4
294	4	1	4	2	2	2	2	3	3	3	3	4	5	1	2	2	3	1	2	1	5	4
296	3	1	2	3	5	4	5	3	3	3	3	4	5	2	3	1	2	1	1	4	4	4
297	4	2	2	1	5	4	5	5	5	5	5	3	3	3	4	4	2	5	5	3	3	4
298	3	1	4	1	4	3	3	3	4	4	2	4	2	2	3	4	4	4	3	4	4	4
299	2	1	2	2	5	3	4	5	4	4	4	4	3	5	4	5	3	5	5	3	2	4
300	3	2	2	3	4	5	5	5	5	4	4	4	4	4	4	5	4	4	4	4	4	4
301	2	1	2	2	5	4	3	4	3	4	3	3	3	3	4	3	4	4	3	4	4	4
302	4	2	4	3	1	1	1	2	4	3	1	5	1	5	5	5	5	5	1	2	2	2
303	4	2	2	3	5	3	5	2	4	3	4	4	5	5	4	4	3	3	3	3	5	4
304	4	2	2	4	5	4	5	3	3	3	4	4	4	1	3	2	2	3	3	4	4	5
305	1	2	1	2	3	4	4	5	4	5	5	4	5	5	4	5	3	3	5	4	2	4
306	4	2	2	4	5	5	5	3	3	3	5	4	5	5	4	4	5	4	4	4	4	4
307	4	1	4	1	6	6	5	7	6	6	5	5	7	6	5	7	4	5	6	7	7	4

308	2	2	2	2	2	3	2	4	4	4	5	4	5	2	3	1	1	2	2	1	5	4
309	2	2	1	2	2	2	2	5	4	4	2	1	1	4	3	5	4	2	1	4	2	3
310	2	1	2	4	5	4	4	4	4	4	5	4	4	1	2	2	1	2	4	4	4	5
311	2	1	1	2	5	5	4	5	5	5	4	4	4	4	5	5	4	3	2	5	3	4
312	4	2	2	3	5	4	5	3	4	4	4	4	4	5	4	4	4	4	3	4	3	5
313	2	1	2	3	4	4	5	3	2	3	5	3	4	4	4	4	4	4	3	3	3	5
314	2	2	1	3	7	6	4	3	2	2	7	6	6	7	7	6	6	4	5	7	7	5
315	2	1	2	2	3	3	3	5	4	5	3	3	3	3	3	3	4	4	4	4	4	4
316	2	2	2	3	3	3	4	4	4	4	4	4	5	5	4	5	3	3	3	5	5	5
317	3	1	1	2	5	5	4	4	4	4	4	4	4	5	4	4	5	5	5	3	3	2
318	2	2	2	2	5	3	5	4	3	3	3	2	3	2	3	3	2	2	2	1	1	4
319	2	2	1	2	5	5	5	5	4	3	5	4	4	4	5	5	1	4	4	5	5	5
320	4	2	1	3	5	3	4	4	5	3	5	4	4	4	3	5	4	1	4	3	5	5
321	2	2	2	2	5	5	3	4	4	4	5	5	3	3	5	5	4	4	4	4	4	4
322	4	1	4	3	5	4	5	4	2	4	4	4	4	4	5	3	2	2	2	4	3	2
323	2	2	1	4	1	3	2	4	5	4	5	5	5	5	4	5	2	3	4	3	3	4
324	4	2	2	3	5	5	6	7	7	5	6	5	7	5	5	4	7	6	7	7	6	4
325	4	1	2	3	4	5	3	5	5	4	5	4	5	5	4	5	5	4	4	4	4	4
326	4	2	3	3	5	4	5	5	4	5	4	4	4	5	4	4	4	2	4	3	3	3
327	3	1	2	3	5	3	4	4	4	4	4	4	4	4	5	5	4	4	4	4	4	3
328	4	2	3	3	5	6	4	5	5	6	7	5	5	6	7	6	7	5	6	5	7	6
329	2	2	4	2	5	5	5	5	5	5	4	4	2	4	3	2	3	3	3	3	3	3
330	2	1	2	2	7	5	5	7	7	4	6	5	7	4	1	2	5	7	7	7	4	6
331	2	1	2	3	6	7	7	6	6	4	5	5	4	7	6	5	7	5	7	5	7	4
332	4	2	2	4	5	6	7	7	5	7	5	6	7	7	7	5	6	5	6	6	6	5
333	4	2	1	2	5	4	5	2	2	4	4	5	5	5	4	4	4	4	4	4	4	4
334	2	2	4	2	5	4	5	5	4	4	4	5	5	5	3	4	5	5	5	3	3	5
335	3	1	2	4	1	1	1	3	4	3	5	5	5	3	3	3	5	5	5	5	5	5
336	4	2	2	3	4	4	5	4	3	5	4	4	5	3	4	4	2	3	4	1	3	5
337	4	1	2	3	5	4	3	5	5	5	4	4	4	4	5	5	5	5	3	5	4	4
338	4	2	2	2	2	2	2	2	3	3	2	2	2	2	2	2	3	1	2	3	3	1
339	4	1	2	3	5	4	5	3	3	3	4	3	5	5	5	5	5	5	5	5	5	4
340	4	2	2	4	5	7	6	7	4	5	4	6	5	4	6	6	7	7	5	7	5	5
341	2	2	3	2	5	5	5	2	3	3	4	5	4	5	5	4	5	5	5	4	4	4
343	4	2	2	4	5	4	5	5	5	4	4	5	4	4	5	5	3	3	3	4	4	3
344	4	2	2	4	4	5	4	5	4	4	4	3	5	3	4	4	4	4	4	3	4	4
345	3	1	2	2	5	6	7	4	7	7	7	5	5	7	7	7	6	7	6	5	6	6
346	4	2	2	2	5	4	5	4	4	3	4	4	5	4	3	4	1	5	5	1	1	1
347	2	1	2	4	5	6	7	7	7	4	7	5	6	5	7	6	7	7	5	6	7	7
348	2	1	2	3	7	7	5	7	5	5	6	5	7	5	7	6	6	6	6	7	6	6
349	2	1	2	2	1	3	1	4	4	4	2	2	1	5	4	4	4	4	4	1	1	5
350	2	1	2	3	4	5	5	5	5	5	5	3	4	4	5	3	3	3	3	5	4	3
351	1	2	2	2	1	3	2	4	3	2	3	2	2	3	2	2	3	3	3	1	1	1
352	2	2	2	3	5	3	5	4	2	4	2	2	3	2	3	3	2	3	2	2	2	2
353	4	1	1	3	4	4	4	3	4	3	4	5	4	2	3	1	3	2	2	2	2	3
354	2	2	3	2	1	2	1	3	1	5	3	2	3	2	3	3	2	2	2	2	2	2
355	4	1	4	1	2	2	2	1	5	2	1	2	2	3	5	5	3	1	2	3	3	1
356	2	2	3	3	1	2	2	3	2	4	3	2	2	4	3	5	2	2	2	2	3	2
357	2	1	2	2	2	2	4	3	3	3	2	3	3	3	3	3	3	4	3	4	4	4
358	3	2	2	3	3	3	3	3	4	4	3	3	3	3	3	3	3	3	3	3	3	3
359	4	2	4	1	4	3	3	3	1	5	3	3	3	3	3	3	3	3	3	3	3	3
360	2	2	2	4	3	5	4	2	4	3	4	5	4	5	4	5	5	5	5	1	1	1
361	2	2	1	3	5	5	5	5	5	4	5	5	5	4	5	4	1	1	4	4	4	4
362	2	1	3	4	2	1	1	5	4	4	3	1	1	3	5	4	4	4	4	2	2	2
363	2	1	4	1	1	2	2	3	3	3	5	5	4	2	2	1	2	2	2	3	3	4
364	2	1	2	2	3	4	4	5	4	4	5	4	4	2	2	1	5	5	5	1	1	1
365	4	1	3	3	5	5	5	5	5	3	2	1	1	4	4	4	5	5	5	1	1	1
366	2	1	2	4	4	5	3	3	3	3	4	3	4	4	3	4	3	3	2	3	3	5
367	2	1	3	3	2	2	1	4	4	4	5	5	4	5	4	4	3	2	2	4	3	4
368	4	1	2	1	4	4	5	3	4	4	4	5	4	4	4	4	2	2	2	4	4	4
369	2	2	2	2	4	4	4	3	2	3	1	2	2	4	4	5	2	3	3	4	4	5
370	4	1	2	3	1	2	1	4	4	4	5	5	5	4	3	5	5	5	5	4	5	1
371	4	1	2	3	6	5	5	5	6	5	5	4	6	7	7	6	6	7	7	6	6	4
372	2	2	2	2	1	3	2	4	3	3	5	5	5	4	5	5	1	3	5	1	5	5
373	4	2	2	3	2	3	1	3	3	4	1	3	2	4	5	5	2	4	4	4	3	4
374	2	2	3	3	5	4	5	3	3	3	4	4	3	4	3	3	4	4	4	2	4	3
375	2	2	3	3	1	2	1	4	5	4	4	5	4	4	4	4	3	5	3	3	3	4
376	2	1	2	2	5	4	5	3	3	3	5	3	4	3	4	4	3	4	3	2	4	5
377	2	2	2	2	5	4	5	3	4	3	3	3	3	5	4	5	5	4	5	5	2	2
378	2	1	2	2	5	4	4	4	4	5	4	5	4	4	5	5	3	4	4	3	3	4
379	2	2	2	4	4	5	4	5	5	4	4	3	3	5	4	5	4	3	3	4	4	5
380	2	2	2	4	5	4	5	5	5	5	3	4	3	4	4	4	3	3	4	5	5	5
381	1	1	2	3	4	3	5	5	5	5	4	4	5	4	4	5	4	5	4	5	4	4
382	4	1	2	4	3	3	4	3	3	3	5	4	4	4	5	3	5	5	3	4	1	5
383	4	1	2	3	4	4	4	5	5	5	4	5	5	5	4	4	5	5	5	3	3	3
384	2	1	2	2	5	5	5	4	5	4	4	5	4	5	5	4	5	5	4	3	3	5

385	2	2	3	4	5	5	3	4	4	3	4	5	5	5	5	4	5	4	5	4	3	5
387	2	1	3	2	4	5	5	7	6	7	5	5	5	4	5	5	5	5	5	5	5	5
388	2	2	2	1	5	5	5	4	5	5	5	5	5	5	5	5	5	5	5	5	5	5
389	2	2	3	4	5	6	7	5	6	7	5	5	7	1	3	2	7	7	7	3	3	3
390	2	2	2	3	4	6	6	7	6	7	7	7	7	7	7	7	2	3	2	2	2	2
391	2	2	2	3	7	7	5	2	4	4	5	7	6	7	5	7	7	6	7	7	5	5
392	4	2	3	3	5	6	6	5	4	7	6	6	6	6	7	5	6	6	5	6	6	6
393	3	2	2	2	5	4	6	3	2	4	5	5	7	6	6	7	6	6	5	5	4	7
394	3	1	3	3	5	5	6	5	6	6	6	7	6	6	5	5	6	6	7	6	5	6
395	4	2	3	4	1	3	2	3	4	3	4	4	4	4	5	5	3	1	1	4	4	4
396	1	1	2	1	6	5	7	4	5	7	3	3	3	5	4	6	7	5	6	6	7	5
397	3	1	2	1	1	2	2	3	3	3	3	1	2	1	3	2	2	3	3	1	1	1
398	4	1	3	4	4	6	6	5	7	6	7	7	7	5	5	6	5	7	7	5	5	4
399	1	1	2	4	6	6	5	7	5	5	5	6	5	7	7	6	2	4	3	6	7	7
400	2	1	2	3	7	6	6	6	7	5	6	4	5	4	7	6	7	4	4	6	5	5
401	4	1	2	3	6	7	4	7	6	7	6	5	6	5	5	4	5	6	5	7	6	7
402	4	1	2	2	6	6	5	7	6	6	7	5	7	5	6	4	7	7	7	7	7	4
403	3	2	2	2	4	4	5	4	4	4	5	5	5	3	5	5	4	4	3	4	3	4
404	1	1	2	3	5	5	3	5	4	5	4	5	5	4	4	5	3	5	5	3	5	5
405	3	2	2	3	3	2	3	3	2	2	1	3	2	4	5	5	2	2	2	3	3	1
406	1	1	2	4	1	1	1	4	3	3	1	5	5	5	5	5	5	5	1	2	2	2
407	2	2	2	3	7	5	6	7	4	7	5	6	6	7	6	6	6	7	7	3	1	4
408	2	2	2	2	2	1	2	4	3	4	5	4	4	1	2	1	3	2	3	2	3	3
409	2	2	2	4	4	4	4	4	5	3	3	5	3	4	3	5	3	1	1	2	3	1
410	4	2	3	3	5	5	5	7	6	7	2	3	2	7	5	6	5	6	7	7	5	5
411	4	2	2	2	3	4	4	4	4	3	5	4	4	5	3	4	3	5	3	3	5	4
412	4	1	2	2	5	4	4	3	5	5	5	4	4	4	5	3	4	4	4	3	5	5
413	4	1	2	3	4	6	7	4	5	5	7	6	7	7	4	7	7	7	4	2	3	4
414	2	2	0	1	4	4	5	5	4	7	5	5	5	4	5	5	4	5	5	5	5	5
415	4	2	3	1	1	1	1	4	3	3	4	4	2	4	4	3	3	4	4	4	4	4
416	4	2	2	4	2	2	2	4	3	3	4	5	5	3	4	4	2	3	4	3	3	4
417	2	1	2	2	5	5	4	6	6	5	5	4	5	5	4	4	5	5	5	5	4	4
